# Short-term efficacy of different chemotherapy regimens in the treatment of advanced gastric cancer: a network meta-analysis

**DOI:** 10.18632/oncotarget.14664

**Published:** 2017-01-14

**Authors:** Mi-Ma Duo-Ji, Ba-Sang Ci-Ren, Zi-Wen Long, Xiao-Hua Zhang, Dong-Lin Luo

**Affiliations:** ^1^ Department of Medicine, Shigatse People's Hospital, Shigatse 857000, P.R. China; ^2^ Department of Gastric Cancer and Soft-Tissue Sarcoma Sugery, Fudan University Shanghai Cancer Center, Shanghai 200032, P.R. China; ^3^ Department of Oncology, Shanghai Medical College, Fudan University, Shanghai 200032, P.R. China

**Keywords:** gastric cancer, fluorouracil, capecitabine, S-1, chemotherapy

## Abstract

**Objective:**

A network meta-analysis was performed to compare the short-term efficacy of different chemotherapy regimens in the treatment of advanced gastric cancer.

**Methods:**

Randomized controlled trials of different chemotherapy regimens for advanced gastric cancer were included in this study. Network meta-analysis combined direct evidence and indirect evidence to evaluate the odds ratio and draw surface under the cumulative ranking curves of different chemotherapy regimens in advanced gastric cancer.

**Results:**

The results of surface under the cumulative ranking curves showed that S-1 and capecitabine regimens were better than fluorouracil. As for multi-drug combination regimens, the disease control rate of cisplatin + capecitabine, docetaxel + cisplatin + fluorouracil and etoposide + cisplatin + capecitabine regimens were relatively better, while fluorouracil + adriamycin + mitomycin regimen was relatively poorer when compared with cisplatin + fluorouracil regimen. Additionally, the overall response ratio of cisplatin + capecitabine, paclitaxel + fluorouracil, docetaxel + cisplatin + fluorouracil and etoposide + cisplatin + fluorouracil regimens were relatively better, while the disease control rate of fluorouracil + adriamycin + mitomycin regimen was relatively poorer when compared with cisplatin + fluorouracil regimen. Furthermore, the results of cluster analysis demonstrated that cisplatin + capecitabine, etoposide + cisplatin + capecitabine, S-1 + paclitaxel and S-1 + irinotecan chemotherapy regimens had better disease control rate and overall response ratio for advanced gastric cancer patients.

**Conclusion:**

This network meta-analysis clearly showed that multi-drug combination chemotherapy regimens based on capecitabine and S-1 might be the best chemotherapy regimen for advanced gastric cancer.

## INTRODUCTION

Gastric cancer (GC) is the 4^th^ most common malignant disease and the 2^nd^ most frequent cause of cancer-related deaths around the world [1]. GC is accepted as a pathophysiologically heterogeneous disease, which is associated with hematogenous metastasis, predominant lymphatic spread, or intra-abdominal spread [2]. There are approximately one million new cases annually worldwide and 850,000 deaths from GC, or about 12% deaths of all cancer [3]. GC is a multifactorial disease caused by environmental and lifestyle factors, other recognized risk factors include smoking, obesity, dietary factors, radiation, Helicobacter pylori infection, pernicious anemia and partial gastrectomy, etc. [4]. Early detection is possible with screening, but most GC patients are diagnosed at an inoperable advanced stage which requiring palliative chemotherapy [5]. The most widely used drugs as single agents for chemotherapy are fluorouracil, doxorubicin, cisplatin, mitomycin C, epirubicin, and etoposide, and newer chemotherapeutic agents include the taxanes, oxaliplatin oral fluoropyrimidines, and irinotecan [6]. Despite the advances achieved over the recent decades, the prognosis of patients with advanced GC (AGC) remains poor [7]. New chemotherapy regimens are desperately needed for the benefit of improving the dismal prognosis of AGC.

Currently, there is no globally accepted chemotherapy regimen for AGC, some are used as single agents, while others are used as part of combination regimens. Fluorouracil is one of the most widely used agents in the treatment of AGC, and it is a part of all the primary multidrug regimens that have been reported [8]. Fluorouracil monotherapy, as a standard treatment for AGC, is associated with manageable toxicity, a response rate of approximately 20%, and OS times of between 5~7 months in phase III randomized studies [9]. Recently, capecitabine and S-1, belonging to oral fluoropyrimidines, are suggested to be more tolerable than fluorouracil; and both of them showed exhibit antitumor activity against AGC [10–12]. Capecitabine (Xeloda, F. Hoffmann–La Roche) is known as an oral fluoropyrimidine, which is designed to mimic a continuous infusion of fluorouracil, and it has shown good response rates in AGC patients when given as monotherapy or in combination with other agents in phase II studies [6]. Study has shown that replacing fluorouracil with capecitabine plus cisplatin avoids the need for continuous infusions and the combinations of two agents have few overlapping toxic effects [13]. S-1 is a novel oral fluoropyrimidine consisting of a tegafur (5-FU prodrug), 5-chloro-2, 4-dihydroxypyridine (the dihydropyrimidine dehydrogenase inhibitor) and potassium oxonate (suppresses the gastrointestinal toxicity of tegafur) [14]. In 2007, one phase III trials in Japan (the JCOG9912 trial) demonstrated that S-1 was not inferior to fluorouracil [15]. The other was the SPIRITS trial, which suggested that the combination therapy of S-1-plus-cisplatin was superior to S-1 monotherapy [12]. Since there are controversies in different studies,, no consensus has been reached on the optimal chemotherapy regimen in the treatment of AGG in terms of single drug chemotherapy regimen and multi-drug combination chemotherapy regimens based on fluorouracil, capecitabine and S-1.

Network meta-analysis is a relatively new statistical technique that gives access to compare both direct and indirect evidence, even when two of the interventions have not been directly compared [16]. Network meta-analysis can summarize randomized clinical trials (RTCs) of several different treatment strategies, and supply point estimates for their association with a given endpoint, together with an estimate of incoherence. Therefore, we performed a network meta-analysis to compare the short-term efficacies of different chemotherapy regimens in the treatment of AGC.

## RESULTS

### Baseline characteristics of included study

**Figure 1:**
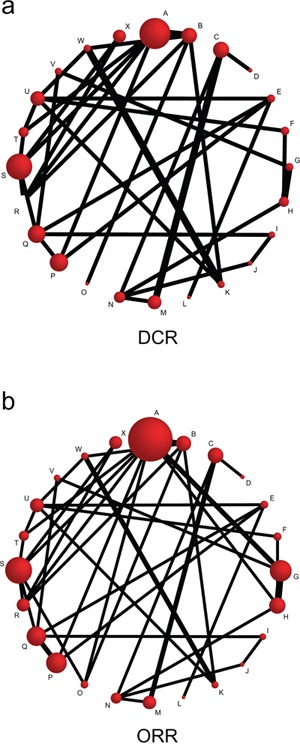
Network diagram for 24 kinds of chemotherapy regimens in terms of DCR and ORR (DCR = disease control rate; ORR = overall response rate; A: cisplatin + fluorouracil; B: fluorouracil; C: S-1; D: capecitabine; E: docetaxel + cisplatin; F: irinotecan + cisplatin; G: cisplatin + capecitabine; H: S-1 + cisplatin; I: docetaxel + fluorouracil; J: paclitaxel + fluorouracil; K: fluorouracil + leucovorin; L: docetaxel + oxaliplatin; M: S-1 + irinotecan; N: S-1 + paclitaxel; O: etoposide + adriamycin + cisplatin; P: docetaxel + cisplatin + fluorouracil; Q: etoposide + cisplatin + fluorouracil; R: fluorouracil + adriamycin + mitomycin; S: fluorouracil + adriamycin + methotrexate; T: etoposide + leucovorin + fluorouracil; U: fluorouracil + leucovorin + irinotecan; V: etoposide + cisplatin + capecitabine; W: fluorouracil + leucovorin + cisplatin; X: cisplatin + etoposide + leucovorin + fluorouracil; a: DCR; b: ORR) The size of the nodes is proportional to the number of studies that evaluate each intervention, and the thickness of the lines is proportional to the frequency of each comparison in the network.

Through electronic databases, 3791 relevant studies were initially identified. We excluded 684 studies for duplicates, 101 for non-human studies, 2420 for no relation to research topic. The remaining 586 articles were further excluded according to the following factors: 124 studies related to targeted therapy, 287 studies related to surgical treatment and 110 studies related to radiotherapy. Eventually, 35 RCTs, published between 1991 and 2014, were eligible for this network meta-analysis [11, 17–50]. These 35 RCTs altogether included 4555 GC patients treated with twenty-four chemotherapy regimens including cisplatin + fluorouracil (CF), docetaxel + cisplatin (DC), irinotecan + cisplatin (CI), cisplatin + capecitabine (CX), S-1 + cisplatin (S-1C), docetaxel + fluorouracil (DF), paclitaxel + fluorouracil (PF), fluorouracil + leucovorin (FL), docetaxel + oxaliplatin (DO), S-1 + irinotecan (S-1I), S-1 + paclitaxel (S-1P), etoposide + adriamycin + cisplatin (EAC), docetaxel + cisplatin + fluorouracil (DCF), etoposide + cisplatin + fluorouracil (ECF), fluorouracil + adriamycin + mitomycin (FAM), fluorouracil + adriamycin + methotrexate (FAMTX), etoposide + leucovorin + fluorouracil (ELF), fluorouracil + leucovorin + irinotecan (FLI), etoposide + cisplatin + capecitabine (ECX), fluorouracil + leucovorin + cisplatin (FLC), and cisplatin + etoposide + leucovorin + fluorouracil (CELF), and the majority of patients received CF and FAMTX chemotherapy regimens (Figure 1a-1b). Of these 35 enrolled studies, 15 studies were from Caucasians, and 20 studies were from Asians; additionally, 29 studies were two-arm trials and 6 studies were three-arm trials. The baseline characteristics of included studies are displayed in Table 1.

**Table 1:** The main baseline characteristics of included studies

Author	Year	Country	Ethnicity	Number			Interventions			Outcomes
				G1	G2	G3	G1	G2	G3	
Wils JA	1991	Netherland	Caucasians	79	81	~	R	S		ORR; DCR
Kelsen D	1992	USA	Caucasians	30	30		O	S		ORR
Kim NK	1993	Korea	Asians	55	54	57	A	B	R	ORR; DCR
Cocconi G	1994	Italy	Caucasians	85	52	~	X	R		ORR; DCR
Waters JS	1999	UK	Caucasians	121	116	~	Q	S		ORR; DCR
Ohkuwa M	2000	Japan	Asians	46	42		A	O		ORR
Vanhoefer U	2000	Netherland	Caucasians	81	85	79	A	S	T	ORR; DCR
Bugat R	2003	France	Caucasians	74	72	~	U	F		ORR; DCR
Cocconi G	2003	Italy	Caucasians	98	97	~	X	S		ORR; DCR
Ohtsu A	2003	Japan	Asians	105	105	~	A	B		ORR; DCR
Bouche O	2004	France	Caucasians	45	45	44	K	U	W	ORR; DCR
Moehler M	2005	Germany	Caucasians	56	58	~	U	T		ORR; DCR
Thuss-Patience PC	2005	Germany	Caucasians	45	45	~	Q	I		ORR; DCR
Park SH	2006	Korea	Asians	39	38	~	J	I		ORR; DCR
Sadighi S	2006	Iran	Asians	42	44		Q	P		ORR
Van CutsemE	2006	Belgium	Caucasians	224	221		A	P		ORR; DCR
Lutz MP	2007	Germany	Caucasians	33	48	46	B	K	W	ORR; DCR
Roth AD	2007	Switzerland	Caucasians	40	41	38	Q	P	E	ORR; DCR
Lee JL	2008	Korea	Asians	45	46		C	D		ORR; DCR
Nakashima K	2008	Japan	Asians	26	36		F	H		ORR; DCR
Popov IP	2008	Serbia	Caucasians	30	30		B	O		ORR; DCR
Kang YK	2009	Korea	Asians	137	139		A	G		ORR
Seol YM	2009	Korea	Asians	32	40		H	G		ORR; DCR
Yun JA	2009	Korea	Asians	44	45		V	G		ORR; DCR
Lim LH	2010	Korea	Asians	37	97	77	A	H	G	ORR
Kim JA	2011	Korea	Asians	28	30		E	U		ORR; DCR
Komatsu Y	2011	Japan	Asians	47	48		C	M		ORR; DCR
Narahara H	2011	Japan	Asians	93	94		C	M		ORR; DCR
Mochiki E	2012	Japan	Asians	41	42		H	N		ORR; DCR
Nishikawa K	2012	Japan	Asians	19	13		N	J		ORR; DCR
Ocvirk J	2012	Slovenia	Caucasians	45	40		Q	V		ORR; DCR
Shitara K	2013	Japan	Asians	37	20		H	G		ORR; DCR
Wang X	2013	China	Asians	41	41		C	N		ORR; DCR
Kim YS	2014	Korea	Asians	38	39		E	L		ORR; DCR
Sugimoto N	2014	Japan	Asians	51	51		N	M		ORR; DCR

Note: G:Group; DCR = disease control rate; ORR = overall response rate; A: cisplatin + fluorouracil; B: fluorouracil; C: S-1; D: capecitabine; E: docetaxel + cisplatin; F: irinotecan + cisplatin; G: cisplatin + capecitabine; H: S-1 + cisplatin; I: docetaxel + fluorouracil; J: paclitaxel + fluorouracil; K: fluorouracil + leucovorin; L: docetaxel + oxaliplatin; M: S-1 + irinotecan; N: S-1 + paclitaxel; O: etoposide + adriamycin + cisplatin; P: docetaxel + cisplatin + fluorouracil; Q: etoposide + cisplatin + fluorouracil; R: fluorouracil + adriamycin + mitomycin; S: fluorouracil + adriamycin + methotrexate; T: etoposide + leucovorin + fluorouracil; U: fluorouracil + leucovorin + irinotecan; V: etoposide + cisplatin + capecitabine; W:fluorouracil + leucovorin + cisplatin; X:cisplatin + etoposide + leucovorin + fluorouracil.

### Pairwise meta-analysis for short-term efficacy of twenty-four chemotherapy regimens in the treatment of advanced gastric cancer

**Figure 2:**
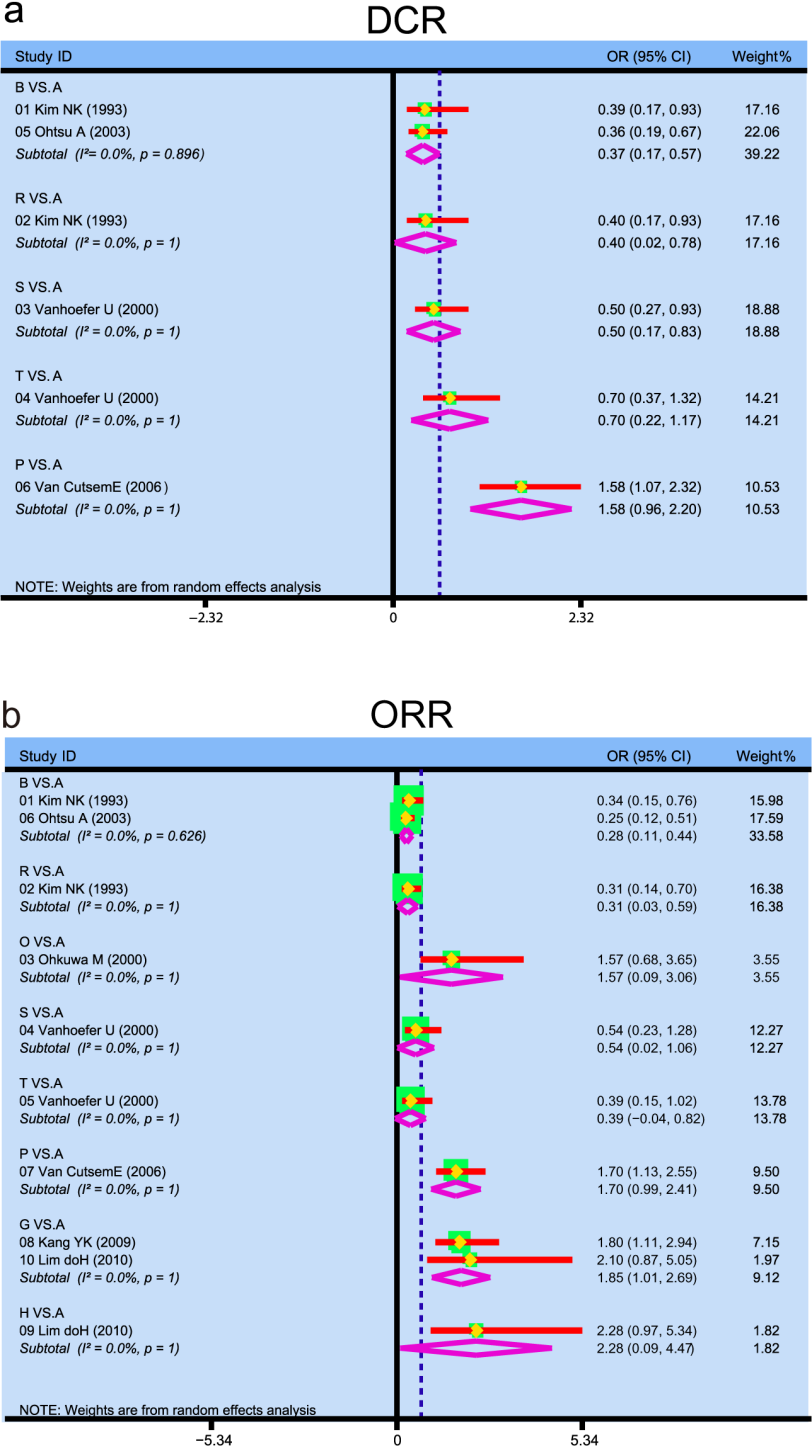
Forest plots of traditional meta-analysis for 24 kinds of chemotherapy regimens in terms of DCR and ORR (DCR = disease control rate; ORR = overall response rate; A: cisplatin + fluorouracil; B: fluorouracil; C: S-1; D: capecitabine; E: docetaxel + cisplatin; F: irinotecan + cisplatin; G: cisplatin + capecitabine; H: S-1 + cisplatin; I: docetaxel + fluorouracil; J: paclitaxel + fluorouracil; K: fluorouracil + leucovorin; L: docetaxel + oxaliplatin; M: S-1 + irinotecan; N: S-1 + paclitaxel; O: etoposide + adriamycin + cisplatin; P: docetaxel + cisplatin + fluorouracil; Q: etoposide + cisplatin + fluorouracil; R: fluorouracil + adriamycin + mitomycin; S: fluorouracil + adriamycin + methotrexate; T: etoposide + leucovorin + fluorouracil; U: fluorouracil + leucovorine + irinotecan; V: etoposide + cisplatin + capecitabine; W: fluorouracil + leucovorin + cisplatin; X: cisplatin + etoposide + leucovorin + fluorouracil; a: DCR; b: ORR)

We carried out direct pairwise comparisons for the short-term efficacies of twenty-four chemotherapy regimens in the treatment of AGC, and the results suggested that the efficacies of fluorouracil, FAM and FAMTX chemotherapy regimens were relatively poorer in DCR of AGC patients when compared with CF regimen (fluorouracil: OR = 0.37, 95%CI = 0.17~0.57; FAM: OR = 0.40, 95%CI = 0.02~0.78; FAMTX: OR = 0.50, 95%CI = 0.17~0.83) (Figure 2a). The efficacies of fluorouracil and FAMTX chemotherapy regimens were relatively poorer in ORR of AGC patients (fluorouracil: OR = 0.28, 95%CI = 0.11~0.44; FAM: OR = 0.31, 95%CI = 0.03~0.59), while CX chemotherapy regimens had better efficacy for AGC (OR = 1.85, 95%CI = 1.01~2.69) (Figure 2b).

### Pooled results of network meta-analysis

#### Inconsistency test of DCR and ORR in included studies

Design-by-treatment interaction model was used for the inconsistency test of DCR and ORR, the Wald test showed that all direct evidence and indirect evidence were consistent, and the fixed effect model was adopted (DCR: *P* = 0.7428, ORR: *P* = 0.2420).

### Comparisons of DCR in different chemotherapy regimens

Totally 30 studies reported the differences of efficacy of DCR in AGC patients treated with twenty-four chemotherapy regimens. Network meta-analysis showed that as for single drug regimen, there was no significant difference for DCR in fluorouracil, S-1, and capecitabine chemotherapy regimens.

**Figure 3:**
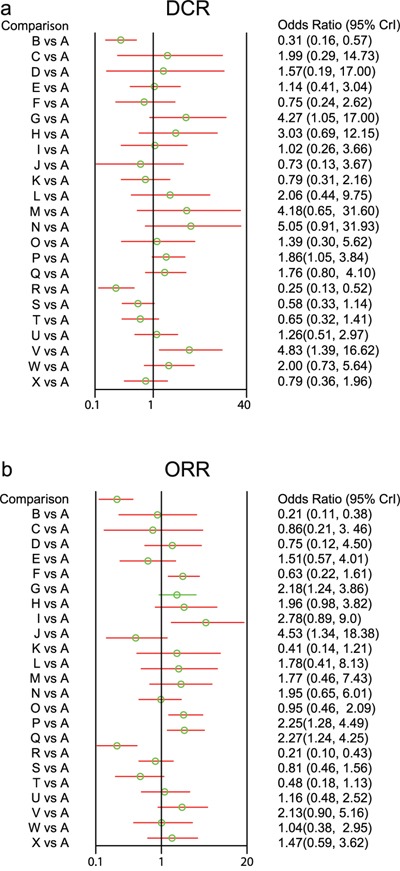
Forest plots of relationship for 24 kinds of chemotherapy regimens in terms of DCR and ORR (DCR = disease control rate; ORR = overall response rate; A: cisplatin + fluorouracil; B: fluorouracil; C: S-1; D: capecitabine; E: docetaxel + cisplatin; F: irinotecan + cisplatin; G: cisplatin + capecitabine; H: S-1 + cisplatin; I: docetaxel + fluorouracil; J: paclitaxel + fluorouracil; K: fluorouracil + leucovorin; L: docetaxel + oxaliplatin; M: S-1 + irinotecan; N: S-1 + paclitaxel; O: etoposide + adriamycin + cisplatin; P: docetaxel + cisplatin + fluorouracil; Q: etoposide + cisplatin + fluorouracil; R: fluorouracil + adriamycin + mitomycin; S: fluorouracil + adriamycin + methotrexate; T: etoposide + leucovorin + fluorouracil; U: fluorouracil + leucovorine + irinotecan; V: etoposide + cisplatin + capecitabine; W: fluorouracil + leucovorin + cisplatin; X: cisplatin + etoposide + leucovorin + fluorouracil; a: DCR; b: ORR)

As for multi-drug combination regimen, AGC patients treated with CX, DCF and ECX chemotherapy regimens had better DCR when compared with CF chemotherapy regimen (CX: OR = 4.27, 95%CI = 1.05~17.00; DCF : OR = 1.86, 95%CI = 1.05~3.84; ECX: OR = 4.83, 95%CI = 1.39~16.62), while the DCR of FAM chemotherapy regimens were relatively poorer (OR = 0.25, 95%CI = 0.13~0.52) (Figure 3a).

Compared with DC chemotherapy regimen, the DCR of ECX chemotherapy regimen was relatively good (OR = 4.03, 95%CI = 1.04~16.78), while the DCR of FAM chemotherapy regimen was relatively poorer (OR = 0.22, 95%CI = 0.07~0.68). The DCR of CX, S-1P and ECX chemotherapy regimens were relatively better than CI chemotherapy regimen (CX: OR = 5.59, 95%CI = 1.40~23.76; S-1P: OR = 6.81, 95%CI = 1.27~42.87; ECX: OR = 6.40, 95%CI= 1.50-26.1). The DCR of CF, CI, PF, FL, FAM, FAMTX, ELF and CELF chemotherapy regimens were relatively poorer than CX chemotherapy regimen (CF: OR = 0.23, 95%CI = 0.06~0.95; CI: OR = 0.18, 95%CI = 0.04~0.71; PF: OR = 0.17, 95%CI = 0.02~0.89; FL: OR = 0.19, 95%CI = 0.04~0.87; FAM: OR = 0.06, 95%CI = 0.01~0.26; FAMTX: OR = 0.14, 95%CI = 0.04~0.55; ELF: OR = 0.16, 95%CI = 0.04~0.67; CELF: OR = 0.19, 95%CI = 0.04~0.90).

Compared with S-1C chemotherapy regimen, the DCR of FAM, FAMTX and ELF chemotherapy regimens were relatively poorer (FAM: OR = 0.08, 95%CI = 0.02~0.41; FAMTX: OR = 0.19, 95%CI = 0.05~0.85; ELF: OR = 0.22, 95%CI = 0.05~0.96). The DCR of ECX chemotherapy regimen was relatively better than DF chemotherapy regimen (OR = 4.54, 95%CI = 1.27~20.81). The DCR of CX, S-1P and ECX chemotherapy regimens were relatively better than PF chemotherapy regimen (CX: OR = 5.90, 95%CI = 1.12~43.52; S-1P: OR = 6.73, 95%CI = 1.09~63.93; ECX: OR = 6.22, 95%CI = 1.31~41.92). Compared with FL chemotherapy regimen, the DCR of CX, S-1P, ECX and FLC chemotherapy regimens were relatively better (CX: OR = 5.21, 95%CI = 1.15~25.77; S-1P : OR = 6.27, 95%CI = 1.02~45.52; ECX: OR = 5.68, 95%CI = 1.43~25.94; FLC: OR = 2.41, 95%CI = 1.12~5.28), while the DCR of FAM chemotherapy regimen was relatively poorer (OR = 0.31, 95%CI = 0.10~0.87).

The DCR of FAM chemotherapy regimen was relatively poorer than DO chemotherapy regimen (OR = 0.12, 95%CI = 0.02~0.60); and the DCR of FAM and FAMTX chemotherapy regimens were relatively poorer than S-1I chemotherapy regimen (FAM: OR = 0.06, 95%CI = 0.01~0.41; FAMTX: OR = 0.14, 95%CI = 0.02~0.91). Compared with S-1P chemotherapy regimen, the DCR of CI, PF, FL, FAM, FAMTX and ELF chemotherapy regimens were relatively poorer (CI: OR = 0.15, 95%CI = 0.02~0.79; PF: OR = 0.15, 95%CI = 0.02~0.92; FL: OR = 0.16, 95%CI = 0.02~0.98; FAM: OR = 0.05, 95%CI = 0.01~0.30; FAMTX: OR = 0.11, 95%CI = 0.02~0.64; ELF: OR = 0.13, 95%CI = 0.02~0.77). The DCR of FAM chemotherapy regimen was relatively poorer than EAC chemotherapy regimen (OR = 0.18, 95%CI = 0.04~0.84). The DCR of CF, FAM, FAMTX and ELF chemotherapy regimens were relatively poorer than DCF chemotherapy regimen (CF: OR = 0.54, 95%CI = 0.26~0.95; FAM: OR = 0.13, 95%CI = 0.05~0.31; FAMTX: OR = 0.31, 95%CI = 0.14~0.67; ELF: OR = 0.35, 95%CI = 0.14~0.83).

Compared with ECF chemotherapy regimen, the DCR of FAM, FAMTX and ELF chemotherapy regimens were relatively poorer (FAM: OR = 0.14, 95%CI = 0.06~0.35; FAMTX: OR = 0.34, 95%CI = 0.17~0.67; ELF: OR = 0.37, 95%CI = 0.15~0.93). Compared with FLI chemotherapy regimen, the DCR of ECX chemotherapy regimen was relatively better (OR = 3.82, 95%CI = 1.06~14.81), while the DCR of FAM chemotherapy regimen was relatively poorer (OR = 0.20, 95%CI = 0.08~0.54). The DCR of FL, FAM and FAMTX chemotherapy regimens were relatively poorer than FLC chemotherapy regimen (FL: OR = 0.41, 95%CI = 0.19~0.89; FAM: OR = 0.13, 95%CI = 0.05~0.38; FAMTX: OR = 0.29, 95%CI = 0.10~0.88). The comparisons of DCR in different chemotherapy regimens are showed in Supplementary Table 1.

### Comparisons of ORR in different chemotherapy regimens

The differences in the ORR of twenty-four chemotherapy regimens in the treatment of AGC were reported in 35 studies. Network meta-analysis revealed that as for single drug regimen, there was no significant difference for ORR in fluorouracil, S-1, and capecitabine chemotherapy regimens. As for multi-drug combination regimen, AGC patients treated with CX, PF, DCF and ECF chemotherapy regimens had better efficacy when compared with CF chemotherapy regimen (CX: OR = 2.18, 95%CI = 1.24~3.86; PF: OR = 4.53, 95%CI = 1.34~18.38; DCF: OR = 2.25, 95%CI = 1.28-4.49; ECF: OR = 2.27, 95%CI = 1.24-4.50), while FAM chemotherapy regimen had poorer efficacy (FAM: OR = 0.21, 95%CI = 0.10~0.43) (Figure 3b)

The ORR of FAM chemotherapy regimen was relatively poorer than DC chemotherapy regimen (OR = 0.14, 95%CI = 0.04~0.44). Compared with CI chemotherapy regimen, the ORR of CX, S-1C, DF, PF, DCF, ECF and ECX chemotherapy regimen was relatively better (CX: OR = 3.49, 95%CI = 1.29~10.46; S-1C: OR = 3.18, 95%CI = 1.20~8.83; DF: OR = 4.44, 95%CI = 1.13~20.04; PF: OR = 7.30, 95%CI = 1.69~38.97; DCF: OR = 3.59, 95%CI = 1.26~12.18; ECF: OR = 3.64, 95%CI = 1.28-11.81; ECX: OR = 3.34, 95%CI = 1.01-12.46). Compared with CX chemotherapy regimen, the ORR of CF, CI, FL, FAM, FAMTX and ELF chemotherapy regimen was relatively poorer (CF: OR = 0.46, 95%CI = 0.26~0.81; CI: OR = 0.29, 95%CI = 0.10~0.78; FL: OR = 0.19, 95%CI = 0.06~0.61; FAM: OR = 0.10, 95%CI = 0.04~0.23; FAMTX: OR = 0.38, 95%CI = 0.18~0.85; ELF: OR = 0.22, 95%CI = 0.07~0.59).

The ORR of CI, FL, FAM and ELF chemotherapy regimens were relatively poorer than S-1C chemotherapy regimen (CI: OR = 0.31, 95%CI = 0.11~0.83; FL: OR = 0.21, 95%CI = 0.07~0.68; FAM: OR = 0.11, 95%CI = 0.04~0.28; ELF: OR = 0.24, 95%CI = 0.08~0.70). The ORR of CI, FL, FAM, FAMTX and ELF chemotherapy regimens were relatively poorer than DF chemotherapy regimen (CI: OR = 0.23, 95%CI = 0.05~0.88; FL: OR = 0.15, 95%CI = 0.03~0.61; FAM: OR = 0.08, 95%CI = 0.02~0.25; FAMTX: OR = 0.30, 95%CI = 0.09~0.91; ELF: OR = 0.17, 95%CI = 0.04~0.65). Compared with PF chemotherapy regimen, the ORR of CF, CI, FL, EAC, FAM, FAMT and ELF chemotherapy regimen were relatively poorer (CF: OR = 0.22, 95%CI = 0.05~0.75; CI: OR = 0.14, 95%CI = 0.03~0.59; FL: OR = 0.09, 95%CI = 0.02~0.43; EAC: OR = 0.21, 95%CI = 0.04~0.96; FAM: OR = 0.05, 95%CI = 0.01~0.18; FAMTX: OR = 0.18, 95%CI = 0.04~0.66; ELF: OR = 0.11, 95%CI = 0.02~0.44).

Compared with FL chemotherapy regimen, the ORR of CX, S-1C, DF, PF, S-1P, DCF, ECF, FLI, ECX and FLC chemotherapy regimens were relatively better (CX: OR = 5.34, 95%CI = 1.65~17.11; S-1C: OR = 4.78, 95%CI = 1.47~15.37; DF: OR = 6.74, 95%CI = 1.65~29.06; PF: OR = 10.94, 95%CI = 2.33~60.45; S-1P: OR = 4.84, 95%CI = 1.01~20.27; DCF: OR = 5.42, 95%CI = 1.85~18.24; ECF: OR = 5.55, 95%CI = 1.84~17.86; FLI: OR = 2.77, 95%CI = 1.08~6.94; ECX: OR = 5.16, 95%CI = 1.39~19.25; FLC: OR = 2.53, 95%CI = 1.15~5.47). The ORR of FAM chemotherapy regimen was relatively poorer than DO chemotherapy regimen (OR = 0.12, 95%CI = 0.02~0.57). The ORR of FAM chemotherapy regimen was also poorer than S-1I chemotherapy regimen (OR = 0.12, 95%CI = 0.03~0.55). Compared with S-1P chemotherapy regimen, the ORR of FL, FAM and ELF chemotherapy regimens were relatively poorer (FL: OR = 0.21, 95%CI = 0.05~0.99; FAM: OR = 0.11, 95%CI = 0.03~0.40; ELF: OR = 0.25, 95%CI = 0.06~0.95). Compared with EAC chemotherapy regimen, the ORR of PF chemotherapy regimen was relatively better (OR = 4.75, 95%CI = 1.04~24.22), while the ORR of FAM chemotherapy regimen was relatively poorer (OR = 0.22, 95%CI = 0.08~0.58). The ORR of CF, CI, FL, FAM, FAMTX and ELF chemotherapy regimens were relatively poorer than DCF chemotherapy regimen (CF: OR = 0.44, 95%CI = 0.22~0.78; CI : OR = 0.28, 95%CI = 0.08~0.79; FL: OR = 0.18, 95%CI = 0.05~0.54; FAM: OR = 0.09, 95%CI = 0.04~0.22; FAMTX: OR = 0.36, 95%CI = 0.17~0.76; ELF: OR = 0.22, 95%CI = 0.07~0.55).

The ORR of CF, CI, FL FAM, FAMTX and ELF chemotherapy regimens were relatively poorer than ECF chemotherapy regimen (CF: OR = 0.44, 95%CI = 0.22~0.81; CI : OR = 0.27, 95%CI = 0.08~0.78; FL: OR = 0.18, 95%CI = 0.06~0.54; FAM: OR = 0.09, 95%CI = 0.04~0.21; FAMTX: OR = 0.35, 95%CI = 0.19~0.68; ELF: OR = 0.21, 95%CI = 0.07~0.53). Compared with FLI chemotherapy regimen, the ORR of FL, FAM and ELF chemotherapy regimens were relatively poorer (FL: OR = 0.36, 95%CI = 0.14~0.93; FAM: OR = 0.19, 95%CI = 0.07~0.53; ELF: OR = 0.41, 95%CI = 0.19~0.97). Compared with FLC chemotherapy regimen, the DCR of FL and FAM chemotherapy regimens were relatively poorer (FL: OR = 0.40, 95%CI = 0.18~0.87; FAM: OR = 0.21, 95%CI = 0.06~0.65). The comparisons of ORR in different chemotherapy regimens are displayed in detail in Supplementary Table 2.

### Surface under the cumulative ranking curves (SUCRA) curves for short-term efficacy of twenty-four chemotherapy regimens in the treatment of advanced gastric cancer

As shown in Table 2, in the outcome of DCR, the chemotherapy regimens of S-1 and capecitabine was better than fluorouracil in terms of single drug regimen, while S-1P chemotherapy regimen had better efficacy for AGC patients with respect to multi-drug combination regimen. In the outcome of ORR, the chemotherapy regimens of S-1 and capecitabine was better than fluorouracil in terms of single drug regimen, while PF chemotherapy regimen had better efficacy for AGC patients with respect to multi-drug combination regimen.

**Table 2:** SUCRA values of twenty four treatment modalities under two endpoint outcomes

Treatment	SUCRA values	
	DCR	ORR
A	0.399	0.393
B	0.051	0.033
C	0.619	0.359
D	0.546	0.330
E	0.450	0.577
F	0.277	0.246
G	0.859	0.763
H	0.756	0.712
I	0.403	0.820
J	0.283	0.934
K	0.307	0.137
L	0.655	0.655
M	0.848	0.666
N	0.900	0.705
O	0.530	0.385
P	0.659	0.767
Q	0.616	0.780
R	0.027	0.040
S	0.184	0.317
T	0.225	0.161
U	0.496	0.477
V	0.896	0.740
W	0.674	0.437
X	0.315	0.577

Notes: DCR = disease control rate; ORR = overall response rate; A: cisplatin + fluorouracil; B: fluorouracil; C: S-1; D: capecitabine; E: docetaxel + cisplatin; F: irinotecan + cisplatin; G: cisplatin + capecitabine; H: S-1 + cisplatin; I: docetaxel + fluorouracil; J: paclitaxel + fluorouracil; K: fluorouracil + leucovorin; L: docetaxel + oxaliplatin; M: S-1 + irinotecan; N: S-1 + paclitaxel; O: etoposide + adriamycin + cisplatin; P: docetaxel + cisplatin + fluorouracil; Q: etoposide + cisplatin + fluorouracil; R: fluorouracil + adriamycin + mitomycin; S: fluorouracil + adriamycin + methotrexate; T: etoposide + leucovorin + fluorouracil; U: fluorouracil + leucovorin + irinotecan; V: etoposide + cisplatin + capecitabine; W: fluorouracil + leucovorin + cisplatin; X: cisplatin + etoposide + leucovorin + fluorouracil.

### Cluster analysis for short-term efficacy of twenty-four chemotherapy regimens in the treatment of advanced gastric cancer

**Figure 4:**
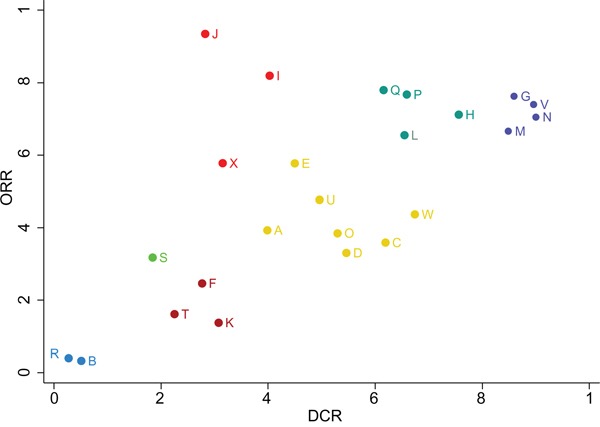
Cluster analysis plots for 24 kinds of chemotherapy regimens in terms of DCR and ORR (DCR = disease control rate; ORR = overall response rate; A: cisplatin + fluorouracil; B: fluorouracil; C: S-1; D: capecitabine; E: docetaxel + cisplatin; F: irinotecan + cisplatin; G: cisplatin + capecitabine; H: S-1 + cisplatin; I: docetaxel + fluorouracil; J: paclitaxel + fluorouracil; K: fluorouracil + leucovorin; L: docetaxel + oxaliplatin; M: S-1 + irinotecan; N: S-1 + paclitaxel; O: etoposide + adriamycin + cisplatin; P: docetaxel + cisplatin + fluorouracil; Q: etoposide + cisplatin + fluorouracil; R: fluorouracil + adriamycin + mitomycin; S: fluorouracil + adriamycin + methotrexate; T: etoposide + leucovorin + fluorouracil; U: fluorouracil + leucovorine + irinotecan; V: etoposide + cisplatin + capecitabine; W: fluorouracil + leucovorin + cisplatin; X: cisplatin + etoposide + leucovorin + fluorouracil)

**Figure 5:**
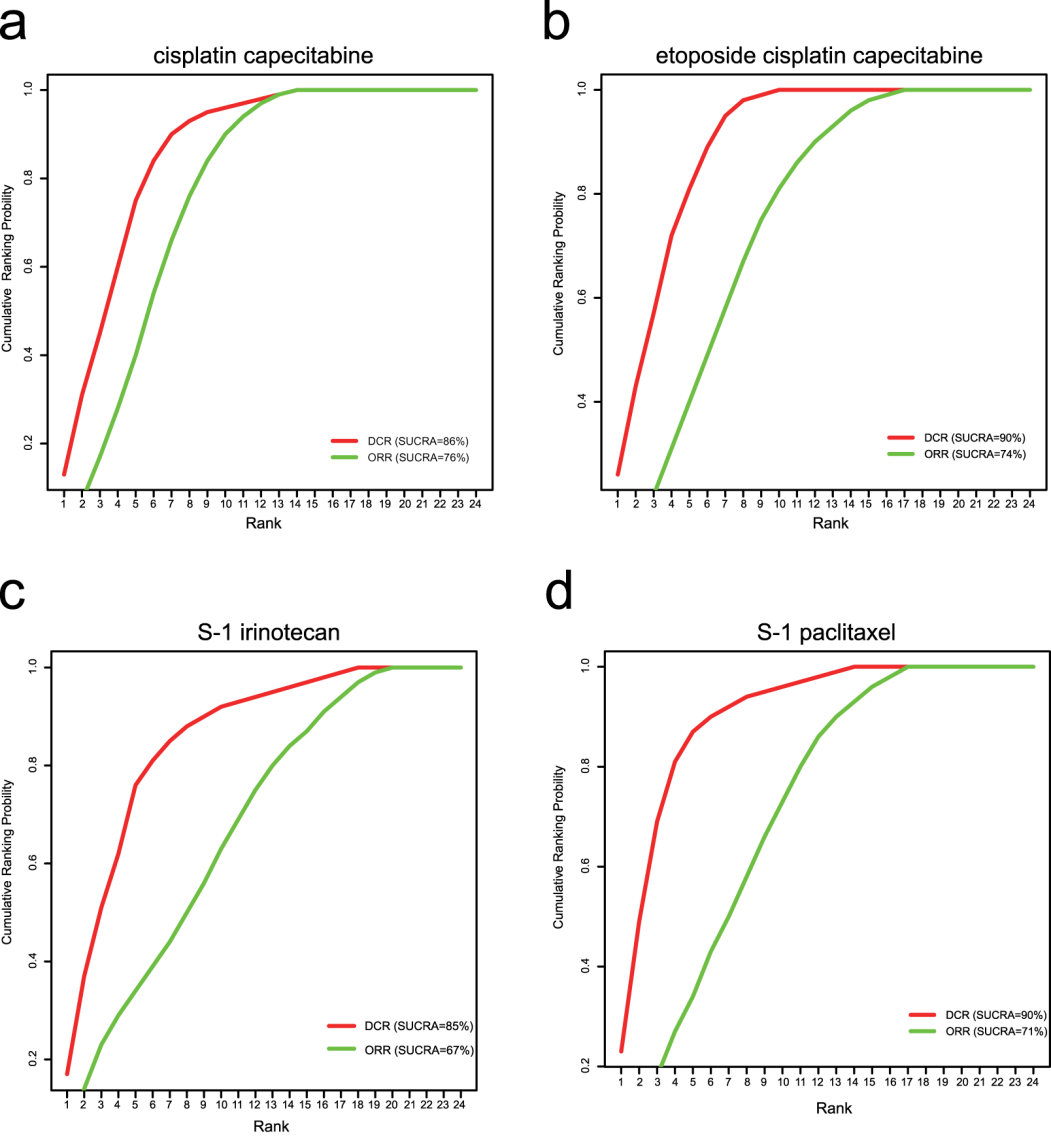
SUCRA plots for chemotherapy regimens of CX, ECX, S-1P and S-1I (a: cisplatin + capecitabine; b: etoposide + cisplatin + capecitabine; c: S-1+ paclitaxel; d: S-1 + irinotecan)

Cluster analysis was conducted for the SUCRA values of twenty-four chemotherapy regimens in the outcomes of DCR and ORR. The results of cluster analysis demonstrated that AGC patients treated with CX, ECX, S-1P and S-1I chemotherapy regimens had better efficacy (Figure 4). The SUCRA plots of CX, ECX, S-1P and S-1I chemotherapy regimens under different outcomes are showed in Figure 5a-5d.

## DISCUSSION

The network meta-analysis results revealed that there was no significant difference for DCR and ORR in the chemotherapy regimens of fluorouracil, S-1 and capecitabine in terms of single drug regimen, while the results of SUCRA showed that the chemotherapy regimens of S-1 and capecitabine were better than fluorouracil. Various chemotherapeutic agents have been used for improving response rate (RR), progression-free survival (PFS), overall survival (OS), and quality of life in patients with AGC [51]. Fluorouracil-based chemotherapy is commonly used for AGC, which has been suggested to have a survival benefit in comparison with the best supportive care [31]. Study has shown that without the inconvenience and complications associated with central venous catheters for fluorouracil, capecitabine maintains a constant level of 5-FU, which is therefore expected to have improved efficacy and tolerability when compared with protracted infusion 5-FU [52]. In the 1990s, S-1, as an oral derivative of 5-FU, was produced for the treatment of GC. As a single agent, S-1 was expected to have a high response rate of 46%, which rapidly established itself as a standard treatment for GC in Japan and was also used widely in clinical practice [32]. The presence of these new generation agents could play important roles in improving patient outcomes, and presenting the access to establish novel chemotherapeutic strategies and personalized medicine, which would help for each individual to select the optimal therapy and dose based on both the tumor and the patient [53]. In consideration of the ORR, OS as well as safety results, J-L Lee in his study provided evidence that patients with AGC could benefit from capecitabine or S-1 monotherapy with minimal adverse events [54]. A subset analysis of the FLAGS trial demonstrated that S-1 seemed to have better efficacy than 5-FU in diffuse type GC [55]. Both capecitabine and S-1 are more tolerable than 5-FU, and has been shown to present antitumor activity against AGC [56]. To some extent, single agent chemotherapy could be regarded as a good and safe first-line treatment for AGC, and it can avoid the compounding effects of other agents when combination therapy is used.

The network meta-analysis clearly shows that multi-drug combination chemotherapy regimens based on capecitabine and S-1 might be the best chemotherapy regimen for AGC. Various attempts have been made since the 1970s, in order to improve the results of chemotherapy through using multi-drug combination chemotherapy regimens. Randomized trials that making comparisons between monotherapy with combination regimens have consistently indicated increased response rates and survival rates in favor of combination regimens [57]. A randomized phase III trial have compared the combinations of fluorouracil with other agents in AGC: infusional 5-FU plus cisplatin (FUP) vs etoposide, LV and 5-FU (ELF) vs 5-FU, doxorubicin and methotrexate (FAMTX) [46]. However, a chemotherapy combination containing infusional 5-FU has the disadvantage of implanting a central venous catheter, and this procedure increases the incidence of subsequent complications, the costs of treatment administration, and the level of discomfort for the patient. The oral fluoropyrimidines (capecitabine and S-1) have shown particular promise, which therefore have the potential to reduce the times of medical examinations related to intravenous injection (i.v.) administration, to reduce the necessary resources for the implantation of the i.v. device, as well as to eliminate catheter-related adverse events [58]. Koizumi W proposed that combined with other promising drugs (eg. cisplatin, irinotecan and taxanes), oral fluoropyrimidine-based combination therapy was suggested to yield good results, and especially, the combination of cisplatin and oral fluoropyrimidines showed high efficacy, which was expected to be a standard therapy for AGC [12]. When S-1 was combined with other cytotoxic drugs, such as irinotecan and cisplatin, it was found to be promising, with relatively favorable safety profiles and response rates of 50 % [59]. A randomized phase III study concerning cisplatin and capecitabine (CX) combination therapy in AGC patients showed that CX produced an ORR of 46% and a median PFS of 5.6 months, which were remarkably better than the poor results with FU/cisplatin (CF) regimen [60]. All these verified that multi-drug combination chemotherapy regimens based on capecitabine and S-1 showed better efficacy than that based on fluorouracil.

Our network meta-analysis offers several important insights: (1) this study is the first network meta-analysis comparing twenty-four chemotherapy regimens in the treatment of AGC, and it bears important clinical implications; (2) we formulated comprehensive search strategy for minimizing possibilities of publication bias; (3) the article referred to both direct and indirect comparisons; (3) the posterior probabilities of SUCRA were utilized to distinguish the slight differences in chemotherapy regimens. However, several limitations of this meta-analysis deserve comment: (1) the presence of missing data in some of the enrolled studies might bias the results; (2) there showed a slight difference in the baseline characteristics of included patients in this network meta-analysis because of different eligible criteria in these studies; (3) since the S-1 agents in enrolled studies were derived from Asian countries, the S-1 regimens are widely used for AGC in Japan [43]. Furthermore, the interethnic differences of capecitabine were observed in genetic changes, which resulting in different efficacy [34]. There might be a certain racial difference, and subgroup analysis could not be carried out for this reason. These limitations might lead to a slight reduction in the validity of our overall results.

The network meta-analysis clearly shows that multi-drug combination chemotherapy regimens based on capecitabine and S-1 might be the best chemotherapy regimen for AGC. However, due to the limitations in our study, our conclusion is needed to be confirmed by a more adequately designed study for future clinical applications.

## MATERIALS AND METHODS

### Literature search

PubMed, Embase, Ovid, EBSCO and Cochrane Library were searched from the inception of each database to July 2015. The search was conducted using the combination of keywords and free words strategy, the terms included “chemotherapy”, “pharmacotherapy”, “cisplatin”, “fluorouracil”, “Capecitabine”, “Docetaxel”, “Paclitaxel”, “Oxaliplatin”, “S-1” and “gastric cancer”, etc. A manual search was also performed for the reference lists of published articles, and literature searches were supplemented by perusing the reference lists of previous meta-analyses.

### Inclusion, exclusion criteria and data extraction

The inclusion criteria included: (1) study design should be randomized controlled trail (RCT); (2) operative methods included chemotherapy agents for the treatment of advanced gastric cancer (AGC) patients; (3) clinically diagnosed AGC patients who only treated with chemotherapy while without any surgical treatments; (4) end outcomes included disease control rate (DCR) and overall response rate (ORR). The exclusion criteria included: (1) studies with insufficient data; (2) non-RCTs, duplicated publications and AGC patients received surgical treatment or radiotherapy. Two reviewers extracted data from the enrolled studies using a specifically designed form. Additionally, a third reviewer was consulted if agreement could not be reached between these two reviewers.

### Statistical analysis

First, we conducted pair-wise meta-analyses of direct evidence by using the random-effects model, which was supplemented with R version 3.2.1 and the Meta package. Second, random-effects network meta-analysis was performed with the gemtc package. Lu and Ades reported that by linking to Open BUGS, network meta-analysis models the relative effects fitting a generalized linear model (GLM) under the Bayesian framework [61]. The relative effects were converted to a probability, which could judge whether a treatment was best or worst, with the surface under the cumulative ranking (SUCRA) curve of each treatment [62] presented as a percentage, ranging from 0%~100% (0%: worst; 100%: best). Based on SUCRA curve, the summary estimates were showed in league tables through ranking the treatments according to priority of the most significant influence on the outcome under consideration [62]. Our command of cluster rank was used in the production of clustered ranking plots in STATA. Outcome1 and outcome2 were regarded as the data variables which containing the SUCRA values for all the treatments in a network. The different colors referred to the estimated clusters, which were used to group the treatments in accordance with their similarity in terms of both outcomes. The concept of design-by-treatment interaction supplied a useful general framework to explore the inconsistency. Particularly, the application of design-by-treatment interactions successfully dealt with complications arising from the multi-arm trials in an evidence network [63]. Lu and Ades also described that a model motivated mainly by loop inconsistency. We provided all models by using mvmeta, which was a Stata (Stata Corp LP. College Station, TX, USA) macro conducted random-effects multivariate meta-regression by using the restricted maximum likelihood [64].

## SUPPLEMENTARY MATERIALS TABLES







## Abbreviations

CF: cisplatin + fluorouracil
DC: docetaxel + cisplatin
CI: irinotecan + cisplatin
CX: cisplatin + capecitabine
S-1C: S-1 + cisplatin
DF: docetaxel + fluorouracil
PF: paclitaxel + fluorouracil
FL: fluorouracil + leucovorin
DO: docetaxel + oxaliplatin
S-1I: S-1 + irinotecan
S-1P: S-1 + paclitaxel
EAC: etoposide + adriamycin + cisplatin
DCF: docetaxel + cisplatin + fluorouracil
ECF: etoposide + cisplatin + fluorouracil
FAM: fluorouracil + adriamycin + mitomycin
FAMTX: fluorouracil + adriamycin + methotrexate
ELF: etoposide + leucovorin + fluorouracil
FLI: fluorouracil + leucovorin + irinotecan
ECX: etoposide + cisplatin + capecitabine
FLC: fluorouracil + leucovorin + cisplatin
CELF: cisplatin + etoposide + leucovorin fluorouracil
