# Supplementary material for: Short-term efficacy of different chemotherapy regimens in the treatment of advanced gastric cancer: a network meta-analysis
Source: Oncotarget. 2017 Jan 14;8(23):37896–911. doi: 10.18632/oncotarget.14664 (PMC5514960; doi:10.18632/oncotarget.14664)
Supplement: Supplementary file 1 [file oncotarget-08-37896-s001.pdf]

Short-term efficacy of different chemotherapy regimens in the treatment of advanced gastric cancer: a network meta-analysis

SUPPLEMENTARY TABLES

Supplementary Table 1: Odds ratio and 95% confidence intervals of twenty four treatment modalities for DCR according to the network meta-analysis

See Supplementary File 1

Supplementary Table 2: Odds ratio and 95% confidence intervals of twenty four treatment modalities for ORR according to the network meta-analysis

See Supplementary File 2
